# Antigen specificity, not tissue compartment, determines clonal sharing among respiratory tract CD8 ^+^ resident memory T cells

**DOI:** 10.1111/imcb.70098

**Published:** 2026-03-02

**Authors:** Thi H O Nguyen, Hayley A McQuilten, Angela Pizzolla, Sneha Sant, Kim L Harland, Jessica Braverman, Katherine Kedzierska, Linda M Wakim

**Affiliations:** ^1^ Department of Microbiology and Immunology The University of Melbourne, at the Peter Doherty Institute for Infection and Immunity Melbourne VIC Australia

**Keywords:** Influenza, resident memory T cells, TCR repertoire

## Abstract

Tissue‐resident memory (Trm) CD8^+^ T cells in the upper and lower respiratory tract play an instrumental role in combatting influenza virus. While CD8^+^ Trm populations in these compartments differ in longevity and developmental requirements, it remains unclear whether they share clonotypes, indicating a common origin, or are seeded by distinct T‐cell clones. Using a mouse model of influenza virus infection, we investigated the T‐cell receptor (TCR) composition of CD8^+^ Trm specific for two immunodominant influenza‐specific CD8^+^ T‐cell epitopes, derived from the nucleoprotein (NP_366‐374_) and polymerase acidic protein (PA_224‐233_). TCRαβ repertoire analysis of NP_366‐374_‐specific CD8^+^ T cells from the nasal mucosa, lung and spleen revealed substantial clonal overlap across resident and circulating subsets, consistent with derivation from a shared precursor pool. In contrast, PA_224‐233_‐specific responses showed minimal clonal sharing across tissues and subsets, reflecting a more diverse and private repertoire. Thus, nasal and lung CD8^+^ Trm can arise from either shared or distinct clonotypes, with their profiles shaped primarily by the antigen specificity rather than tissue location. This allows barrier tissues to be seeded by both highly conserved, public NP_366–374_‐specific CD8^+^ TCRs and a more diverse, private PA_224–233_‐specific repertoire, together forming an optimal pool capable of providing broadly cross‐reactive T‐cell immunity and limiting viral escape.

## Graphical abstract

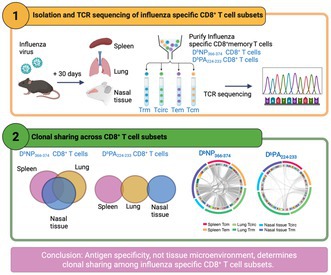

## INTRODUCTION

Influenza virus infections remain a major global public health threat, causing annually a billion infections, 3–5 million severe cases and 290 000–650 000 deaths.^1^ Disease severity is closely linked to the site of viral infection. When viral infection is restricted to the upper respiratory tract, symptoms are generally mild–moderate (coughing, sneezing, fatigue). However, influenza can disseminate into the lower respiratory tract, where viral infection of the lungs can result in life‐threatening and potentially fatal disease.

Cytotoxic CD8^+^ T cells play a key role in controlling influenza virus infection by eliminating virus‐infected cells. Among these, a specialized subset of memory CD8^+^ T cells, termed tissue‐resident memory T cells (Trm), occupy the upper and lower respiratory tract and are indispensable for protective immunity.^2^, ^3^ Trm are nonrecirculating, self‐sustaining memory T cells lodged within peripheral tissues that provide local protection against reinfection.^4^ Previous studies have shown that waning protection against pulmonary influenza virus infection correlates with the loss of influenza‐specific lung Trm.^3^ Moreover, we and others have demonstrated that boosting influenza‐specific CD8^+^ Trm in the nasal mucosa can block viral spread into the lower respiratory tract, preventing severe disease and associated pathology.^2^, ^5^, ^6^, ^7^, ^8^ Nasal Trm also reduce viral shedding, thereby lowering transmissibility^7^, ^9^ and rendering animals less contagious.^7^, ^9^

Although both are critical for respiratory immunity, CD8^+^ Trm in the upper and lower airways display distinct characteristics. For example, nasal CD8^+^ Trm are longer‐lived than their lung counterparts and develop independently of TGFβ signaling and local cognate antigen recognition, whereas lung CD8^+^ Trm require both TGFβ and local antigen recognition for establishment.^2^, ^4^ It remains unclear whether these fundamental differences in development and longevity arise from seeding by distinct T‐cell clones with predetermined fates, or whether nasal and lung CD8^+^ Trm are derived from a common precursor, with local tissue cues shaping their development and persistence.

Using a mouse model of influenza virus infection, we examined the longevity, functional avidity and T‐cell receptor (TCR) repertoire of nasal tissue and lung CD8^+^ Trm specific for two immunodominant influenza‐specific epitopes, derived from nucleoprotein (NP_366‐374_) and polymerase acidic protein (PA_224‐233_). Overall, our findings indicate that despite differences in longevity and developmental requirements, nasal and lung Trm can be derived from either shared or distinct clonotypes, driven by antigen specificity rather than tissue microenvironment.

## RESULTS AND DISCUSSION

### Memory CD8 ^+^ T cells persist in the upper respiratory tract after influenza virus infection with minimal numerical decay

**Figure 1:**
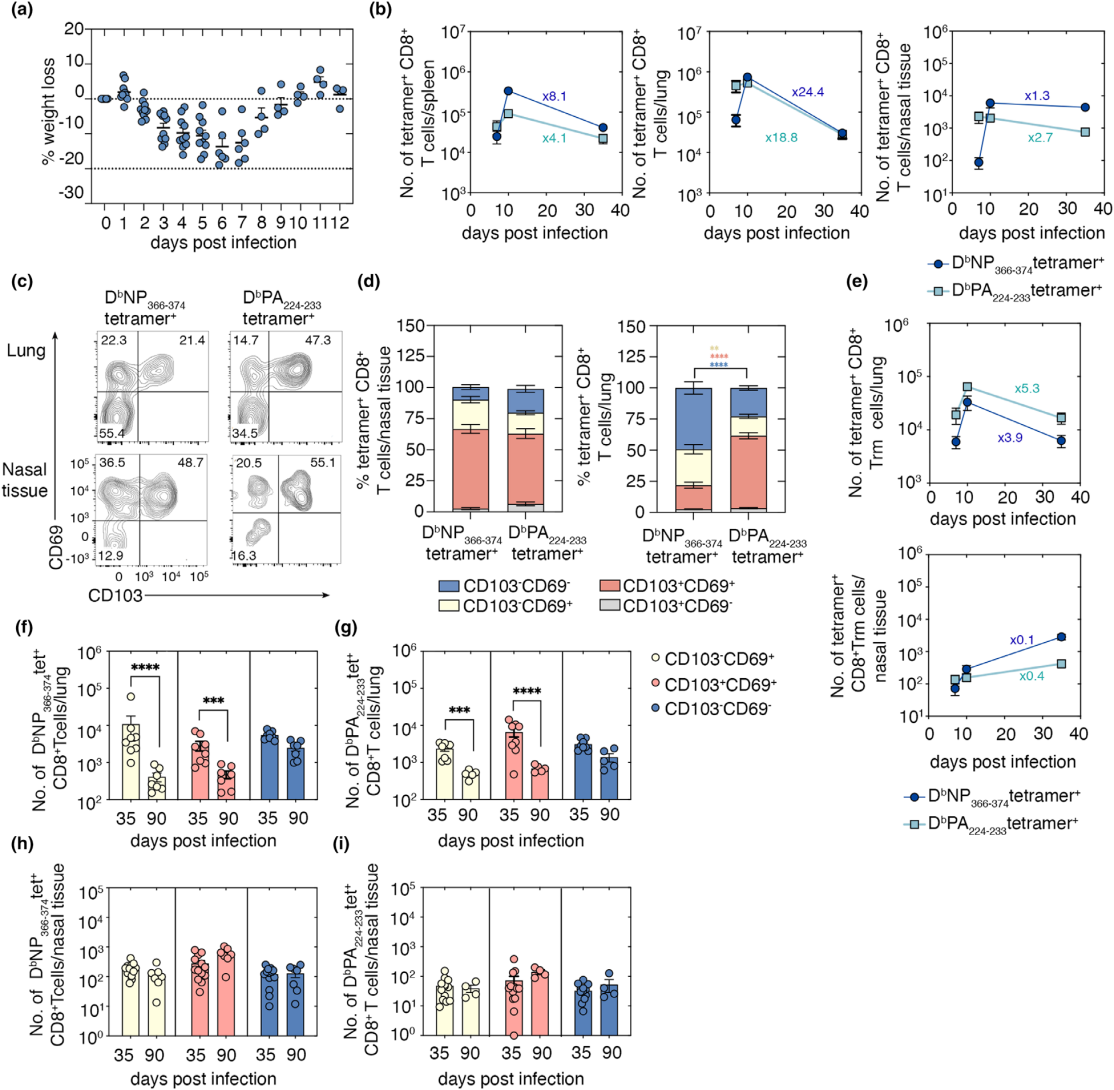
Memory CD8^+^ T‐cell responses in upper and lower respiratory tract after influenza virus infection. **(a)** Percentage weight loss of mice infected with 10^4^ PFU of X31 and monitored until weight returned to pre‐infection levels. Symbols represent individual mice and bars represent the mean ± SEM (*n* = 4, or 6 or 10 mice per time point). **(b)** Absolute numbers of D^b^NP_366_‐_374_ and D^b^PA_224_‐_233_ specific CD8^+^ T cells in the spleen, lung and nasal tissue of mice infected with 10^4^ PFU of X31 were determined at days 7, 10 and 35 postinfection (p.i.). Data are pooled from 2 experiments and symbols represent means ± SEM (*n* = 8–9 mice per group). Numbers inserted in the graphs represent fold change reduction in cell numbers between d10 and d35 postinfection. **(c)** Flow cytometry plots and **(d)** mean percentage expression of CD69 and CD103 on D^b^NP_366_‐_374_ and D^b^PA_224_‐_233_‐specific CD8^+^ T cells in the lung and nasal tissue of mice infected with 10^4^ PFU of X31 and analyzed on d35 p.i. Bars represent the mean ± SEM (*n* = 7 or 8 mice per group; two‐way ANOVA followed by Sidak's multiple comparison). **(e)** Absolute number of CD103^+^ CD69^+^ D^b^NP_366_‐_374_ and D^b^PA_224_‐_233_‐specific CD8^+^ T cells in the lung and nasal tissue on days 7–35 p.i. are shown. Data are pooled from two experiments, and symbols represent individual mice and bars show the means ± SEM (*n* = 8–9 mice per group). **(f–i)** Absolute number of CD103^+^CD69^+^, CD103^−^CD69^–^ and CD103^−^CD69^+^ **(f, h)** D^b^NP_366_‐_374_ and **(g, i)** D^b^PA_224_‐_233_ specific CD8^+^ T cells in the **(f, g)** lung and **(h, i)** nasal tissue on days 35 and 90 p.i. with 10^4^ PFU of X31. Data are pooled from two experiments, and symbols represent individual mice and bars show means ± SEM (*n* = 7–8 mice per group; two‐way ANOVA followed by Sidak's multiple comparison).

To define the establishment and maintenance of CD8^+^ tissue‐resident memory (Trm) cells across the respiratory tract, we used a well‐established intranasal influenza A virus infection mouse model that causes moderate disease (Figure 1a) and generates Trm in both the upper and lower airways.^2^ C57BL/6 mice were infected intranasally with 10^4^ PFU of influenza A virus, X31, and CD8^+^ T cells specific for two immunodominant influenza‐specific CD8^+^ T‐cell epitopes, D^b^NP_366‐374_ and D^b^PA_224‐233_ were quantified in the spleen, lung and nasal tissue. The number and Trm differentiation of these virus‐specific CD8^+^ T cells, defined by CD69 and +/− CD103 expression, were evaluated at the acute (day 7 and 10), early memory (day 35) and late memory (day 90) time points postinfection (p.i.). D^b^NP_366‐374_‐ and D^b^PA_224‐233_‐specific effector CD8^+^ T‐cell responses peaked at day 7–10 p.i. across all tissues (Figure 1b). Between days 10 and 35 p.i., an 18‐24‐fold contraction of D^b^NP_366‐374_‐ and D^b^PA_224‐233_‐specific CD8^+^ T cells occurred in the lung, whereas influenza virus‐specific CD8^+^ T cell numbers in the nasal tissue remained relatively stable over this period (Figure 1b). Phenotypic analysis at day 30 p.i. showed that, consistent with previous findings,^2^ the proportion of D^b^PA_224‐233_‐specific CD69^+^CD103^+^ Trm was higher than D^b^NP_366‐374_‐specific Trm in the lung, while in the nasal tissue both specificities differentiated equivalently into CD69^+^CD103^+^ Trm (Figure 1c, d). Longitudinal analysis revealed that in the lung, both CD69^+^CD103^−^ and CD69^+^CD103^+^ D^b^NP_366‐374_‐ and D^b^PA_224‐233_‐specific resident memory CD8^+^ T cells declined significantly between days 35 and 90 p.i. while CD69^−^CD103^−^ cells, likely representing circulating memory T cells confirmed by their labelling following intravenous antibody staining (Supplementary figure 1), remained stable (Figure 1e–g). In contrast, both CD69^+^CD103^−^ and CD69^+^CD103^+^ D^b^NP_366‐374_‐ and D^b^PA_224‐233_‐specific resident memory CD8^+^ T‐cell subsets in the nasal tissue were stably maintained over the same period (Figure 1e, h, i). These data demonstrate that, consistent with prior reports, nasal CD8^+^ Trm exhibit increased longevity compared with lung CD8^+^ Trm.

### Memory CD8 ^+^ T cells in the upper respiratory tract after influenza virus infection have reduced TCR biases compared to lung memory CD8 ^+^ T cells

**Figure 2:**
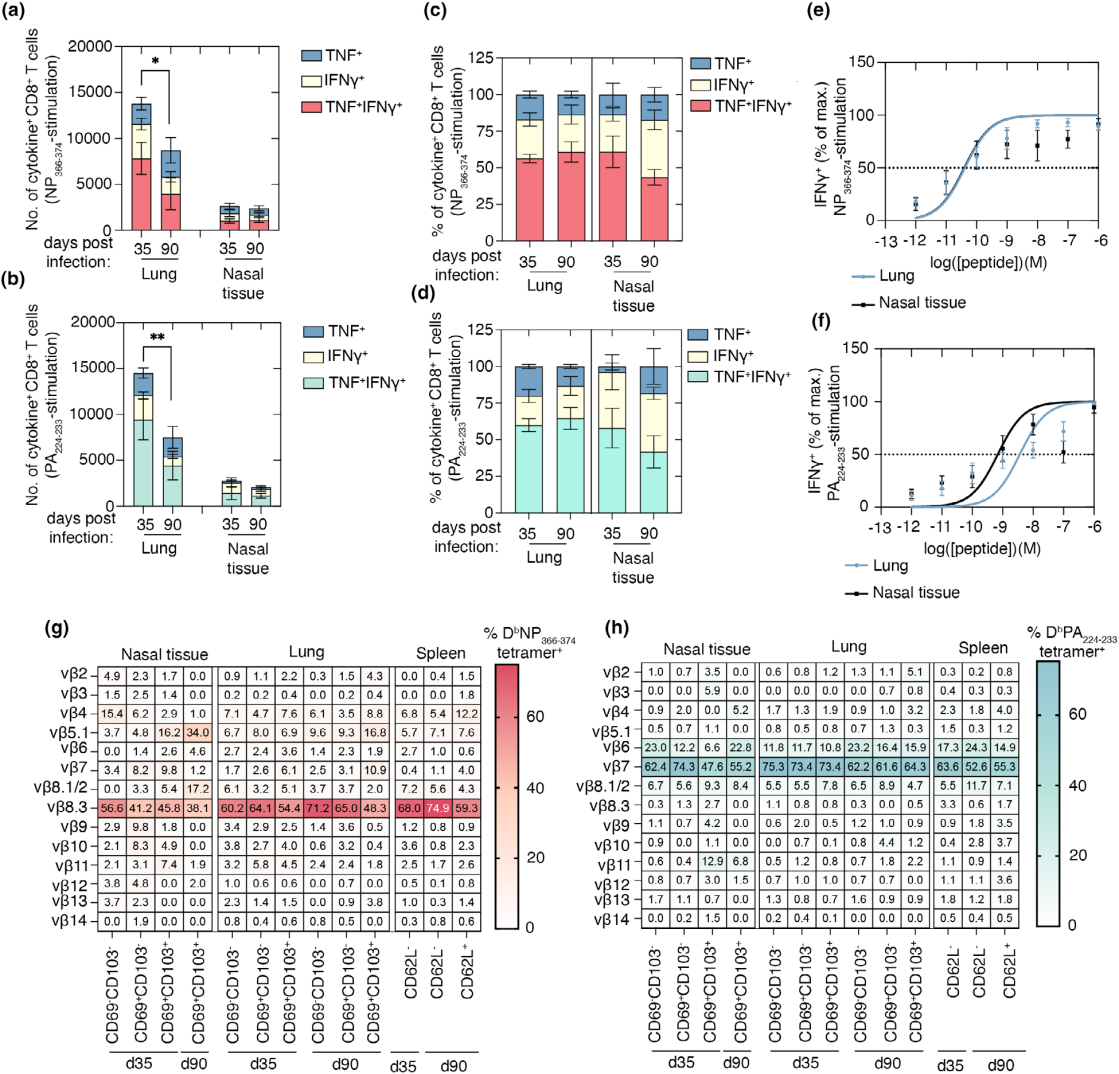
Assessment of the TCR biases and polyfunctionality in upper and lower respiratory tract memory CD8^+^ T cells. **(a, b)** Absolute number and **(c, d)** percentage of IFNγ^+^, TNF^+^ and IFNγ/TNF^+^ double‐producing CD8^+^CD44^+^ T cells isolated from the lung and nasal tissue of mice that were infected with 10^4^ PFU of X31 35 or 90 days prior, following either (a, c) NP_366_‐_374_ peptide or (b, d) PA_224_‐_233_ peptide stimulation. Data are pooled from two experiments and bars represent the mean ± SEM (*n* = 5; two‐way ANOVA with Šidák's multiple comparisons). **(e, f)** Peptide titration curves of the percentage max of IFNγ^+^ production of bulk memory CD8^+^CD44^+^ T cells isolated from the lung and nasal tissue of mice infected with influenza virus 35 days prior. Cells were restimulated with graded concentrations of (e) NP_366‐374_ peptide or (f) PA_224‐233_ peptide. Dotted line represents the 50% maximum response. **(g, h)** Normalized frequencies of Vβ TCR usage for CD103^+^CD69^+^, CD103^−^CD69^−^, and CD103^−^CD69^+^ (g) D^b^NP_366‐374_‐ or (h) D^b^PA_224‐233_‐specific CD8^+^ T cells in the lung, nasal tissue and CD62L+ and CD62L‐ spleen cells recovered from mice 35‐ and 95‐days postinfection with 10^4^ PFU of X31. Data are pooled from three experiments [(*n* = 2–4 samples (pooled tissue from 2 to 3 mice/sample)].

To assess how the quality of CD8^+^ Trm in the upper and lower respiratory tract changes over time, we examined their cytokine production and TCR avidity. C57BL/6 mice were infected intranasally with 10^4^ PFU of influenza A virus, X31, and lung, spleen and nasal tissues were collected on days 35 and 90 p.i. The frequency of IFNγ and TNF single producers, as well as IFNγ^+^/TNF^+^ double‐producing CD8^+^ memory T cells, was measured following a brief *in vitro* stimulation with either NP_366‐374_ or PA_224‐233_ peptides in the presence of brefeldin A. Although the overall number of NP_366‐374_‐ or PA_224‐233_‐specific cytokine‐producing CD8^+^ T cells in the lung declined significantly between days 35 and 90 p.i. (Figure 2a, b), the relative proportion of TNF or IFNγ single producers and IFNγ^+^/TNF^+^ double producers remained stable (Figure 2c, d), indicating that surviving lung‐resident memory cells maintained their polyfunctionality despite their numerical contraction. In contrast, while the total frequency of NP_366‐374_ or PA_224‐233_‐specific cytokine‐producing CD8^+^ T cells in the nasal mucosa remained stable over this period (Figure 2a, b), there was a decline, although not statistically significant, in the proportion of IFNγ^+^/TNF^+^ double producers, suggesting a potential reduction in polyfunctional CD8^+^ memory T cells in this region (Figure 2c, d). We next compared the antigen sensitivity of nasal versus lung CD8^+^ T‐cell populations. Cells isolated from mice day 35 after influenza infection were stimulated with graded concentrations of NP_366‐374_ or PA_224‐233_ peptide, and IFNγ production was used to determine TCR sensitivity. NP_366‐374_‐specific CD8^+^ memory T cells in the lung and nasal tissue displayed equivalent antigen sensitivity, with similar IFNγ responses across peptide doses (Figure 2e). PA_224‐233_‐specific CD8^+^ T cells exhibited lower antigen sensitivity compared to NP_366‐374_ specific CD8^+^ T cells and this varied across respiratory tissues (Figure 2f). In the nasal mucosa, PA_224‐233_‐specific CD8^+^ T cells exhibited a 5.2‐fold higher antigen sensitivity than their lung counterparts, based on the peptide concentration required to reach 50% maximal IFNγ production (EC_50_) (average LogEC50 lung = −8.440 vs nasal tissue = −9.163) (Figure 2f). It has been reported that lung Trm differentiation is inversely correlated with the strength of TCR signaling, with lower‐affinity clones showing a competitive advantage in adopting a resident phenotype, a mechanism thought to maintain antigen‐specific diversity within tissues.^10^ Consistent with this model, we find that PA_224–233_‐specific CD8^+^ T cells exhibit lower functional avidity than the NP_366–374_‐specific population, particularly within the lung microenvironment. This reduced avidity profile aligns with our observation that a disproportionately greater fraction of PA_224–233_‐specific T‐cell clones differentiate into lung Trm compared with their NP_366‐374_‐specific counterparts (Figure 1b). Together, these findings indicate that influenza‐specific memory CD8^+^ T cells in the nasal tissue and lung differ in antigen sensitivity and potentially polyfunctionality and further support previous findings^10^ that lower‐affinity populations are preferentially channeled into the lung Trm lineage.

### Profiling vβ TCR usage of influenza‐specific CD8 ^+^ T‐cell memory across tissue and time

As the next step, we examined whether differences in antigen sensitivity across lung and nasal tissue‐resident NP_366‐374_‐ and PA_224‐233_‐specific memory CD8^+^ T cells were associated with altered TCRαβ repertoire diversity. We profiled by flow cytometry TCR Vβ usage in D^b^NP_366‐374_‐ and D^b^PA_224‐233_‐tetramer^+^ memory CD8^+^ T‐cell subsets isolated from the lung, nasal tissue and spleen of C57BL/6 mice at days 35 and 90 following intranasal infection with influenza virus (X31). Consistent with previous reports, D^b^NP_366‐374_‐specific CD8^+^ T cells were biased toward Vβ8.3 usage^11^, ^12^, ^13^ although the degree of skewing varied by tissue, time point and subset (Figure 2g). At day 35 p.i., 41.2–45.8% of nasal D^b^NP_366‐374_‐specific CD69^+^CD103^−^ or CD69^+^CD103^+^ CD8^+^ Trm were Vβ8.3^+^, while the double‐negative population showed higher usage (56.6%). This contrasted with the lung D^b^NP_366‐374_‐specific CD8^+^ T‐cell subsets at day 35 p.i., where both CD69^+^CD103^−^ and CD69^+^CD103^+^ Trm subsets exhibited a stronger bias toward Vβ8.3 usage (54.4–64.1%), matching the level of skewing in the lung D^b^NP_366‐374_‐specific double‐negative population and the spleen effector memory T‐cell pool (Figure 2g). By day 90 postinfection, the Vβ8.3 bias slightly decreased within the D^b^NP_366‐374_‐specific CD69^+^CD103^+^ Trm subsets in both lung (less 6.1%) and nasal tissue (less 7.7%). At this time point, nasal and lung D^b^NP_366‐374_‐specific CD69^+^CD103^+^ Trm exhibited < 50% Vβ8.3 usage (Figure 2g), whereas circulating D^b^NP_366‐374_‐specific memory T cells in the lung maintained the Vβ8.3 skewing, closely resembling splenic D^b^NP_366‐374_‐specific effector memory cells. It should be noted that at day 90 p.i. only CD103^+^CD69^+^ Trm CD8^+^ T cells were present at sufficient numbers for TCR Vβ profiling in the nasal tissue. For D^b^PA_224‐233_‐specific cells, Vβ7 and Vβ6 dominance was evident across all lung subsets and time points, resembling D^b^PA_224‐233_‐specific effector and central memory CD8^+^ T cells in the spleen (Figure 2h). In contrast, nasal CD69^+^CD103^+^ PA_224‐233_‐specific Trm showed a less polarized Vβ7 bias at both day 35 and 90 postinfection. Reduced Vβ skewing in nasal Trm was not due to increased representation of alternative Vβ families unique to this site, but rather to the expanded presence of Vβ elements that were also found in other compartments (Figure 2h). Collectively, these data indicate that nasal Trm populations retain a less biased TCR repertoire compared with their lung and splenic counterparts, potentially highlighting broader clonal diversity in this barrier tissue.

### Profiling of influenza‐specific CD8 ^+^ T‐cell memory clonotypes across tissue

**Figure 3:**
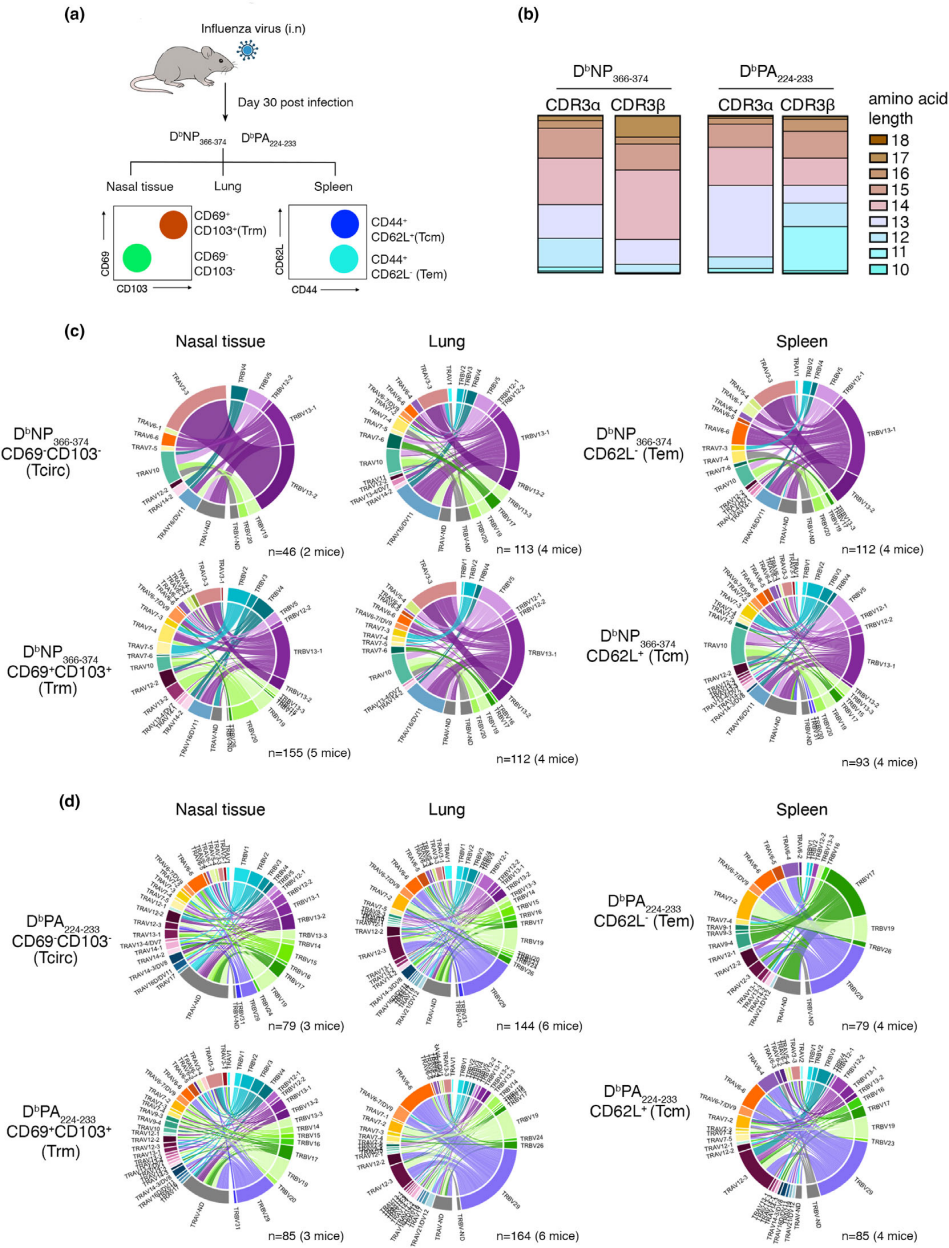
TCRαβ repertoire profiling of D^b^NP_366‐374_‐specific and D^b^PA_224‐233_‐specific memory T‐cell subsets across tissue. **(a)** Influenza virus‐specific memory CD8^+^ T cells (CD8^+^CD44^+^) isolated from lung, spleen and nasal tissue of mice infected 30 days prior with 10^4^ PFU of X31 were single‐cell sorted based on expression of CD103, CD69 and CD62L into Trm (CD103^+^CD69^+^), Tcirc (CD103^−^CD69^−^), Tem (CD62L^−^) and Tcm (CD62L^+^) populations. TCR analysis across 2 prominent influenza‐specific epitopes (D^b^NP_366‐374_ and D^b^PA_224‐233_) was assessed according to their **(b)** CDR3α and CDR3β length. **(c, d)** Circos plots of frequencies of Vβ‐Jβ (TRBV) and Vα‐Jα (TRAV) usage for paired TCRαβ sequences are shown for (c) D^b^NP_366‐374_‐ and (d) D^b^PA_224‐233_‐specific Trm, Tcirc, Tem and Tcm populations. Outer arch segment colored by TRAV and TRBV usage. TRAV–TRBV gene pairing indicated by connecting lines which are colored based on their TRBV usage and segmented based on their CDR3α and CDR3β sequence. The thickness is proportional to TCR clone number with the respective pair. The number at the right bottom of the circos plot indicates the number of sequences considered. Circos plots were generated with the circlize v.0.4.16 software package.

To define the TCR repertoire of nasal, lung and splenic D^b^NP_366‐374_‐ and D^b^PA_224‐233_‐specific CD8^+^ T cells in greater depth, we performed paired TCRαβ sequencing. C57BL/6 mice were infected intranasally with 10^4^ PFU of influenza A virus (X31), and at day 30 p.i. D^b^NP_366‐374_‐ and D^b^PA_224‐233_‐tetramer^+^ CD8^+^ T cells were purified from the lung, spleen and nasal tissue (Figure 3a). Nasal and lung populations were further divided into tissue‐resident (Trm; CD103^+^CD69^+^) and circulating (Tcirc: CD103^−^CD69^−^) subsets, while splenic memory CD8^+^ T cells were separated into effector (Tem; CD62L^−^) and central memory (Tcm; CD62L^+^) subsets. In total, we generated paired TCRαβ data for 631 NP_366‐374_‐specific and 636 PA_224‐233_‐specific CD8^+^ memory T cells isolated from seven mice, representing four phenotypically defined subsets across nasal, lung and splenic tissues (Supplementary figure 2).

As expected, CD8^+^ T cells recognizing distinct epitopes exhibited characteristic biases in TRBV and TRAV usage, as well as differences in CDR3α and CDR3β loop lengths (Figure 3b–d). Assessment of TCR sequences from all mice revealed that the repertoire of D^b^NP_366‐374_‐specific CD8^+^ T cells across tissues and memory T‐cell subsets were restricted and enriched for public TCRβ signatures. TRBV13‐1 usage dominated (magenta purple bands) with CDR3β loops of 14 aa, and typically paired with CDR3α loops of 13–14 aa with variable TRAV use (Figure 3b, c), consistent with previous reports.^13^, ^14^, ^15^ In contrast, the D^b^PA_224‐233_‐specific CD8^+^ TCRαβ repertoire was more diverse, though still showed preferential use of TRBV29 (iris purple bands) and TRBV19 (pale green bands) gene segments with CDR3β lengths of 11 aa and paired with various TRAV segments with CDR3α lengths of 13 aa (Figure 3b, d).

**Figure 4:**
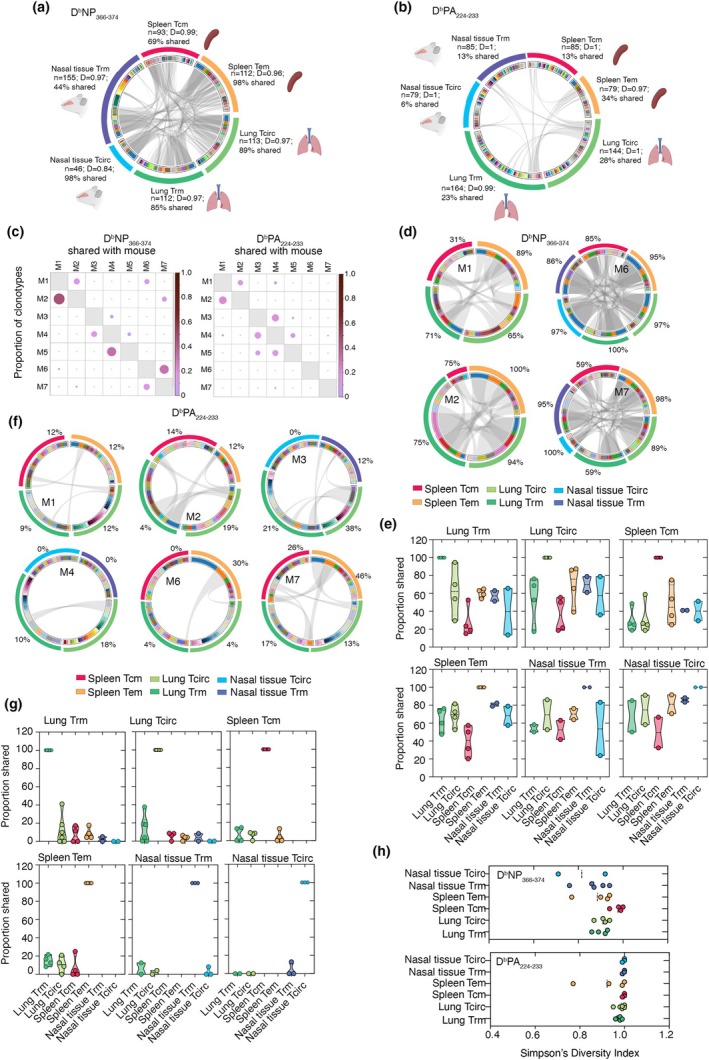
TCRαβ repertoire sharing across T‐cell subsets and tissue. **(a, b)** Circos plots constructed from TCRαβ sequences from all mice pooled showing clonotype sharing between T‐cell subsets and tissues for (a) D^b^NP_366‐374_‐ and (b) D^b^PA_224‐233_‐specific CD8^+^ T cells. Shown are the number of sequences considered and the proportion of clonotypes within each T‐cell subset shared within any other subset. **(c)** Proportion of clonotype sharing between mice for all sequences by color gradient and size of circle. **(d)** Circos plots of clonotype sharing for D^b^NP_366‐374_‐specific Trm, Tcirc, Tem and Tcm populations separated into individual mice. **(e)** Proportion of D^b^NP_366‐374_ CD8^+^ T‐cell clonotype sharing across different T‐cell subsets with other compartments. Symbols represent individual mice. **(f)** Circos plots of clonotype sharing for D^b^PA_224‐233_‐specific Trm, Tcirc, Tem and Tcm populations separated into individual mice. **(g)** Proportion of D^b^PA_224‐233_ clonotype sharing across different T‐cell subsets with other compartments. Symbols represent individual mice. **(h)** Simpson's diversity index (SDI) analysis of clonotypes in D^b^NP_366‐374_ and D^b^PA_224‐233_ epitopes CD8^+^ T cells. Symbols represent individual mice.

To assess the relationship between tissue‐resident and circulating memory CD8^+^ T‐cell populations, we examined paired TCRαβ signatures across nasal and lung Trm and Tcirc subsets, as well as splenic Tem and Tcm. When all sequences from all mice were pooled by subset, > 80% of D^b^NP_366‐374_‐specific T‐cell clonotypes in nasal Tcirc, lung Trm, lung Tcirc and spleen Tem were shared with at least one other memory T‐cell subset (Figure 4a). NP_366‐374_‐specific nasal Trm and splenic Tcm contained a higher proportion of unique clonotypes; however, a substantial fraction (44% and 69%, respectively) was still shared with another subset (Figure 4a). In contrast, performing the same pooled analysis on the D^b^PA_224‐233_‐specific CD8^+^ T‐cell populations revealed only minimal clonal overlap between subsets (6–34%), with the majority of clonotypes being unique to their respective tissue or subset and not present in any other compartment (Figure 4b).

We next assessed whether D^b^NP_366‐374_‐ and D^b^PA_224‐233_‐specific T‐cell clonotypes were shared between individual mice and found minimal inter‐mouse sharing (Figure 4c). This indicated that mouse‐to‐mouse variability could bias pooled analyses. To address this, we repeated the clonotype‐sharing analysis within individual mice. For D^b^NP_366‐374_‐specific CD8^+^ T‐cell populations, four mice were analyzed. Consistent with the pooled analysis, the splenic Tcm subset again exhibited a low degree of clonotype sharing with all other T‐cell subsets on a per‐mouse basis (range 31–85%) (Figure 4d, e; Supplementary figure 3). Nasal Trm displayed extensive sharing, with 86–95% of their clonotypes detected in at least one other subset, while lung Trm clonotype sharing ranged from 59 to 100% (Figure 4d, e; Supplementary figure 3). Assessment of the two mice (M6 and M7), for which complete lung, nasal tissue and spleen T‐cell subset datasets were available, again showed extensive sharing of D^b^NP_366‐374_‐specific CD8^+^ T‐cell clonotypes across all tissues and subsets (Supplementary figure 4). In contrast, analysis of D^b^PA_224‐233_‐specific CD8^+^ T‐cell clonotypes within individual mice again revealed minimal clonal overlap (range 0–49%), with the vast majority of clonotypes unique to each tissue and subset (Figure 4f, g; Supplementary figure 5). Simpson's diversity index analysis revealed comparable clonal diversity across all T‐cell subsets within both D^b^NP_366‐374_‐ and D^b^PA_224‐233_‐specific CD8^+^ T‐cell populations (Figure 4h), suggesting that TCRαβ diversity is maintained across all memory T‐cell compartments. Together, these data demonstrate that D^b^NP_366‐374_‐specific CD8^+^ memory T cells are dominated by a restricted set of public clonotypes and show extensive overlap across resident and circulating T‐cell subsets and between tissues, consistent with derivation from a common precursor pool. Conversely, D^b^PA_224‐233_‐specific CD8^+^ T‐cell responses are predominantly private, exhibiting minimal clonal sharing across subsets or anatomical sites. These findings align with our earlier antigen sensitivity analyses, in which D^b^NP_366‐374_‐specific CD8^+^ T cells displayed similar functional sensitivity in the lung and nasal tissue, consistent with their high degree of clonal overlap. In contrast, the differences in antigen sensitivity observed among DᵇPA_224–233_‐specific CD8^+^ T cells between these tissues are consistent with their derivation from more diverse, tissue‐distinct clonotype pools.

Our study reveals that the degree of clonal sharing among respiratory tract CD8^+^ Trm is dictated primarily by antigen specificity rather than by the anatomical site or T‐cell subset. The D^b^NP_366‐374_‐specific CD8^+^ T‐cell response was dominated by a small number of public clonotypes and exhibited extensive clonal overlap across resident and circulating subsets and across all tissues examined. This pattern is consistent with derivation from a shared precursor pool and aligns with the previously described highly restricted and public D^b^NP_366‐374_‐specific CD8^+^ T‐cell repertoire^12^, ^13^ These results indicate that nasal and lung Trm targeting this epitope are seeded from a common precursor pool and subsequently shaped by local microenvironments, rather than arising from compartment‐specific progenitors. In contrast, D^b^PA_224‐233_‐specific CD8^+^ T‐cell responses were markedly more polyclonotypic and showed minimal clonal sharing between tissues and subsets, reflecting their more diverse and largely private TCR repertoire.

Together, these data show that the clonal relatedness of respiratory Trm populations is not governed by the tissue compartment but is instead a function of epitope‐driven T‐cell repertoire constraints. The capacity of NP_366‐374_‐specific CD8^+^ T‐cell responses to generate broadly shared, high‐quality public clonotypes enables both the upper and lower airways to be populated with clonally overlapping Trm. By contrast, the PA_224‐233_‐specific CD8^+^ T‐cell response populates these same sites with diverse and largely private clonotypes, potentially broadening recognition breadth at the cost of reduced clonal connectivity. This dual strategy, combining optimal, public NP_366‐374_‐specific TCRs with a heterogeneous PA_224‐233_‐specific TCR repertoire, may represent an evolutionarily advantageous arrangement that both maintains strong antiviral defense and limits viral escape.

## METHODS

### Mice and viruses

All experiments were done in accordance with the Institutional Animal Care and Use Committee guidelines of the University of Melbourne (20006, 1714190). C57BL/6 (CD45.2) mice were bred in‐house under specific pathogen‐free conditions in the animal facility at the Peter Doherty Institute of Infection and Immunity, University of Melbourne, Melbourne, Australia. Mice were infected intranasally in a volume of 30 μL with 10^4^ PFU of Hong Kong/31 (H3N2) X31 influenza A virus. Virus was grown in 10‐day‐old embryonated eggs by standard procedures and titrated on Madin‐Darby canine kidney (MDCK) cells, and infection was tracked as previously described.^5^

### Flow cytometry

Single‐cell suspensions were prepared from spleens by mechanical disruption. Mice were perfused before the harvest of the lung and nasal tissue. The nasal tissue, including the nasal cavity, nasal turbinates and NALTs, was obtained by cutting down the vertical plane of the skull and scraping out the tissues and small bones from both sides of the nasal passages. Lung and nasal tissue were enzymatically digested for 1 hr at 37°C in 3 mL of collagenase type 3 (3 mg/mL in RPMI 1640 (Gibco) supplemented with 2% FBS). Virus‐specific CD8^+^ T cells were identified using in‐house produced tetrameric complexes of H2‐D^b^ and the NP_366‐374_ peptide (ASNENMETM) or PA_224‐233_ peptide (SSLENFRAYV).^14^ Cells were incubated with anti‐mouse CD16/32 (Fc block, clone 93, BioLegend) for 10 mins at room temperature, then stained with PE‐conjugated D^b^NP_366‐374_ and APC‐conjugated D^b^PA_224‐233_ tetramers for 1 hr at room temperature, washed with EDTA‐BSS with 2% FCS and then stained with the appropriate mixture of mAbs for 30mins on ice. For intracellular cytokine analysis, single‐cell suspensions of the lung, nasal tissue and spleen were stimulated with the indicated concentration of NP_366‐374_ or PA_224‐233_ peptide for 4 hr at 37°C/10% CO_2_ in the presence of GolgiPlug (BD Biosciences) in complete RPMI (10% FCS, 2 mM glutamine, 50 mM 2‐ME, Pen/Strep) prior to surface staining for 30 mins on ice with the appropriate mixture of mAbs and then intracellularly stained using a Foxp3 fix/perm kit (Thermo Fisher) according to manufacturer's protocol. For peptide titration curves, the % max of IFN‐γ^+^ production of memory CD8^+^CD44^+^ T cells stimulated with graded concentrations of NP_366‐374_ or PA_224‐233_ peptide were used to calculate the corresponding EC_50_ values. Data were normalized to the proportion of cytokine^+^ cells at saturating concentration (10^−6^ M). A nonlinear regression curve was fitted to calculate the EC_50_. The conjugated mAbs obtained from BD Pharmingen or BioLegend include the following: anti‐CD8 (53–6.7), anti‐CD8 (YTS‐169.4), anti‐CD3 (17A2), anti‐CD44 (1 M7), anti‐CD103 (2E7), anti‐CD69 (H1.2F3), anti‐IFNγ (XMG1.2), anti‐TNFα (MP6‐XT22), anti‐CD62L (MEL‐14). Vβ usage analysis was performed using a BD Mouse Vβ TCR Screening Panel Kit (BD Biosciences, San Diego, CA, USA) according to the manufacturer's instructions. Samples were acquired using a Becton Dickinson Fortessa flow cytometer and data were analyzed using the FlowJo software package (Tree Star, Inc., Ashland, OR USA). Representative gating strategies are shown in Supplementary figure 6.

### Anti‐CD8 antibody labelling

Mice were injected intravenously with 3 μg of phycoerythrin‐conjugated antibody to CD8 (clone YTS‐169) 5 min before they were sacrificed. Mice were cardiac perfused with PBS, and tissues were collected, processed and stained with allophycocyanin‐conjugated antibody to CD8 (clone 53–6.7).

### Flow cell sorting and TCR sequencing

NP_366‐374_‐specific and PA_224‐233_‐specific memory CD8^+^ T‐cell subsets were index single‐cell sorted from the lung, nasal tissue and spleen (Trm = CD8^+^CD44^+^CD103^+^CD69^+^; Tcirc = CD8^+^CD44^+^CD103^−^CD69^−^; Tem = CD8^+^CD44^+^CD62L^−^; Tcm = CD8^+^CD44^+^CD62L^+^) of 7 mice on day 30 postinfection with 10^4^ PFU of influenza virus (X31) on a BD Aria III (BD Biosciences) into 96‐well Twin.tec PCR plates (Eppendorf). Paired CDR3α and CDR3β regions of mRNA were amplified by multiplex‐nested reverse‐transcriptase PCR before sequencing of TCRα and TCRβ products and analyzed by IMGT/V‐QUEST, as described.^16^ PCR amplified products were visualized on a gel before sequencing. Cells with low quality of both alpha and beta sequences that could not be aligned to a V gene were excluded. Of the 631 NP clonotypes, 40 were excluded (6%) and of the 636 PA clonotypes none were excluded. In total, we generated paired TCR data for 631 NP_366‐374_ and 636 PA_224‐233_ ‐specific CD8^+^ memory T cells (Supplementary Table 1). Circos plots were generated using circlize v.0.4.16.^17^

### Statistical analysis

Comparison between two study groups was statistically evaluated by unpaired two‐tailed *t*‐test or Mann–Whitney test. Comparison between more than two groups (single factor) was evaluated using one‐way analysis of variance (ANOVA) with Tukey's multiple comparison. Two‐way ANOVA with Šidák's multiple comparison was used to evaluate more than two groups at different time points or cohorts. In all tests, statistical significance was quantified as *P < 0.05, **P < 0.01, ***P < 0.001 and ****P < 0.0001. Statistical analysis was performed using GraphPad v10.

## AUTHOR CONTRIBUTIONS

**Thi H. O. Nguyen:** Investigation; data curation. **Hayley A. McQuilten:** Investigation; data curation. **Angela Pizzolla:** Conceptualization; investigation; data curation. **Sneha Sant:** Data curation. **Kim L. Harland:** Data curation. **Jessica Braverman:** Data curation. **Katherine Kedzierska:** Conceptualization; funding acquisition; supervision. **Linda M. Wakim:** Conceptualization; investigation; funding acquisition; writing – original draft; supervision; data curation; writing – review and editing.

## CONFLICT OF INTEREST

The authors have declared that no conflict of interest exists.

## ETHICS STATEMENT

All experiments were done in accordance with the Institutional Animal Care and Use Committee guidelines of the University of Melbourne (20006, 1714190).

## Supporting information


Supplementary figure 1.
**Supplementary figure 2**.
**Supplementary figure 3**.
**Supplementary figure 4**.
**Supplementary figure 5**.
**Supplementary figure 6**.

Supplementary Table 1.

## Data Availability

The data that support the findings of this study are available from the corresponding author upon reasonable request.
