# Supplementary material for: Antigen specificity, not tissue compartment, determines clonal sharing among respiratory tract CD8 + resident memory T cells
Source: Immunol Cell Biol. 2026 Mar 2;104(4):358–67. doi: 10.1111/imcb.70098 (PMC13071122; doi:10.1111/imcb.70098)
Supplement: Supplementary file 1 — Supplementary figure 1. Supplementary figure 2. Supplementary figure 3. Supplementary figure 4. Supplementary figure 5. Supplementary figure 6. [file IMCB-104-358-s002.docx]

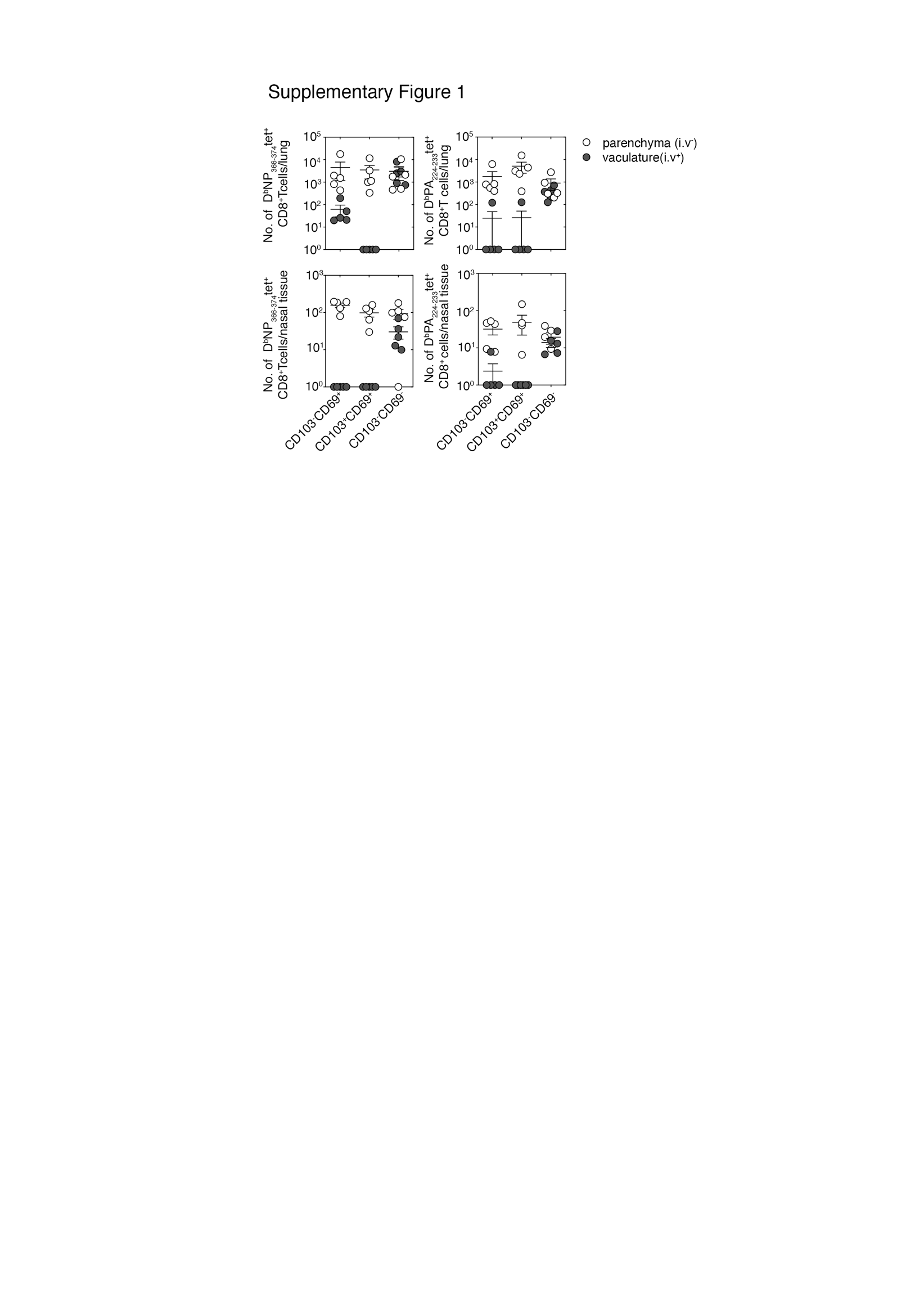


**Supplementary Figure 1: Intravascular antibody staining confirming CD103^+^CD69^+^ CD8^+^ T cell localisation to the parenchyma.**

B6 mice infected with 10^4^ PFU of influenza virus (X31) 35 days prior were injected intravenously with anti-CD8-PE. The absolute number of vasculature associated (i.v. antibody positive) or parenchyma bound (i.v. antibody negative) CD103^+^CD69^+^, CD103^-^CD69^-,^ and CD103^-^CD69^+^ D^b^NP_366_ and D^b^PA_244_- specific CD8^+^ T cells in the lung and nasal tissue was measured. Data pooled from 2 experiments (n = 10).


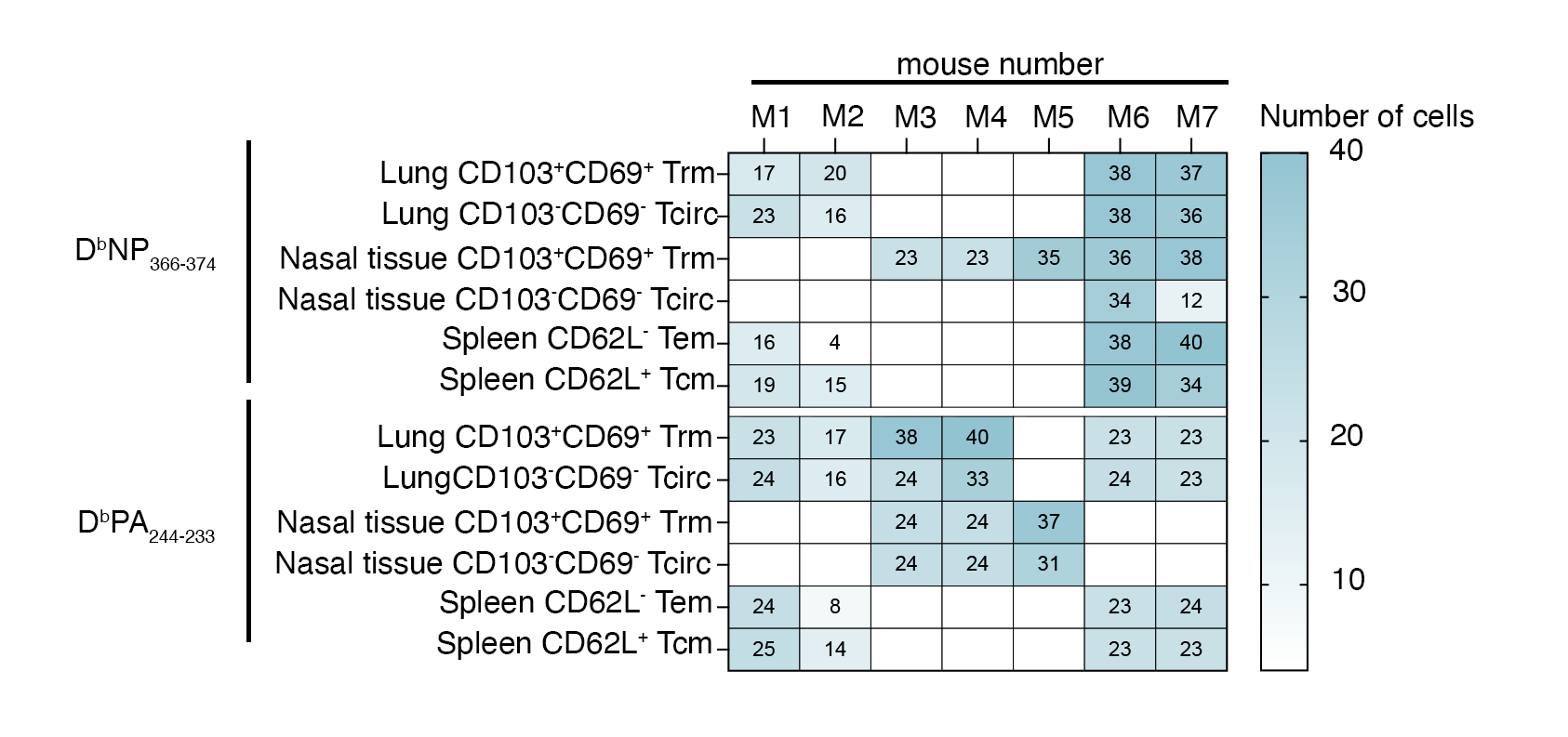


**Supplementary Figure 2: TCRαβ repertoire profiling across T cell subset and tissue.**

Heat map depicting number of D^b^NP_366-374_ and D^b^PA_224-233_ specific CD8^+^ memory T cells sequenced per subset, per tissue and per mouse.


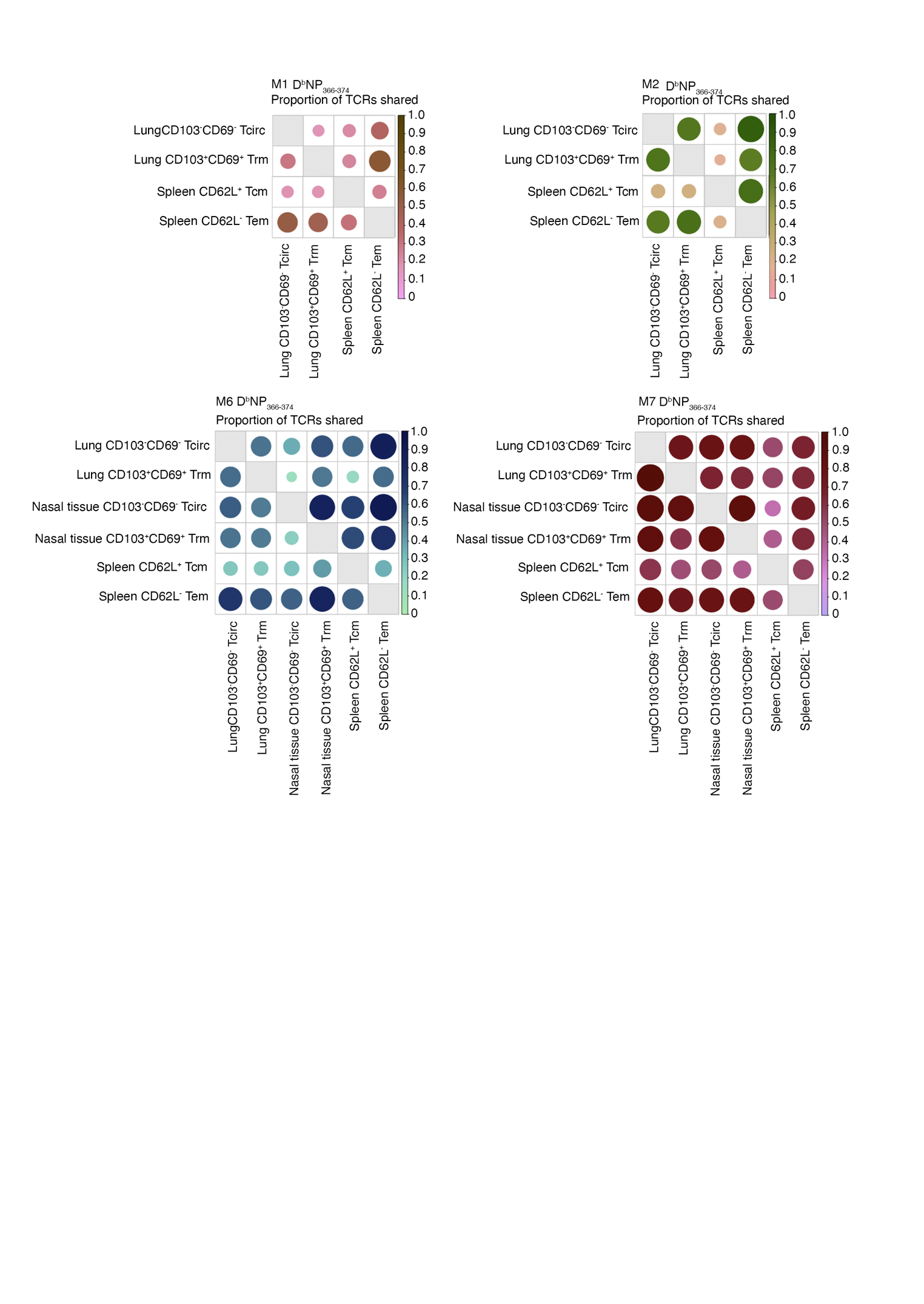


**Supplementary Figure 3:** D^b^**NP_366-374_ specific CD8^+^ T cell TCRαβ clonotype sharing across T cell subset and tissue.**

Proportions of shared clonotypes between D^b^NP_366-374_ tetramer^+^ memory CD8^+^ T cells subsets within individual mice by colour gradient and size of circle.


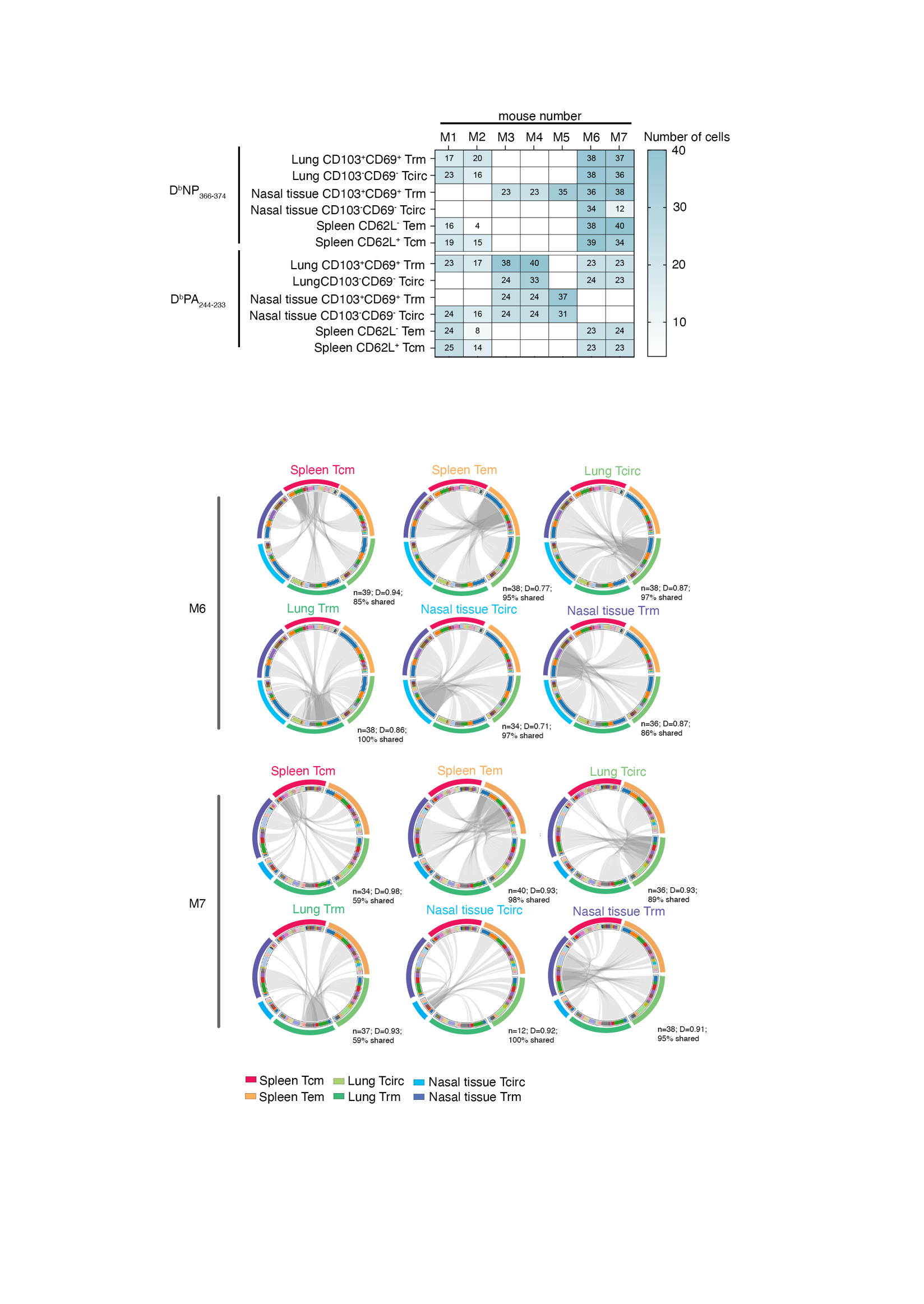


**Supplementary Figure 4:** D^b^**NP_366-374_ specific CD8^+^ T cell TCRαβ clonotype sharing across T cell subset and tissue.**

Circos plots of mouse 6 (M6) and 7 (M7) anchored at different subsets showing percentage of clonotype sharing.


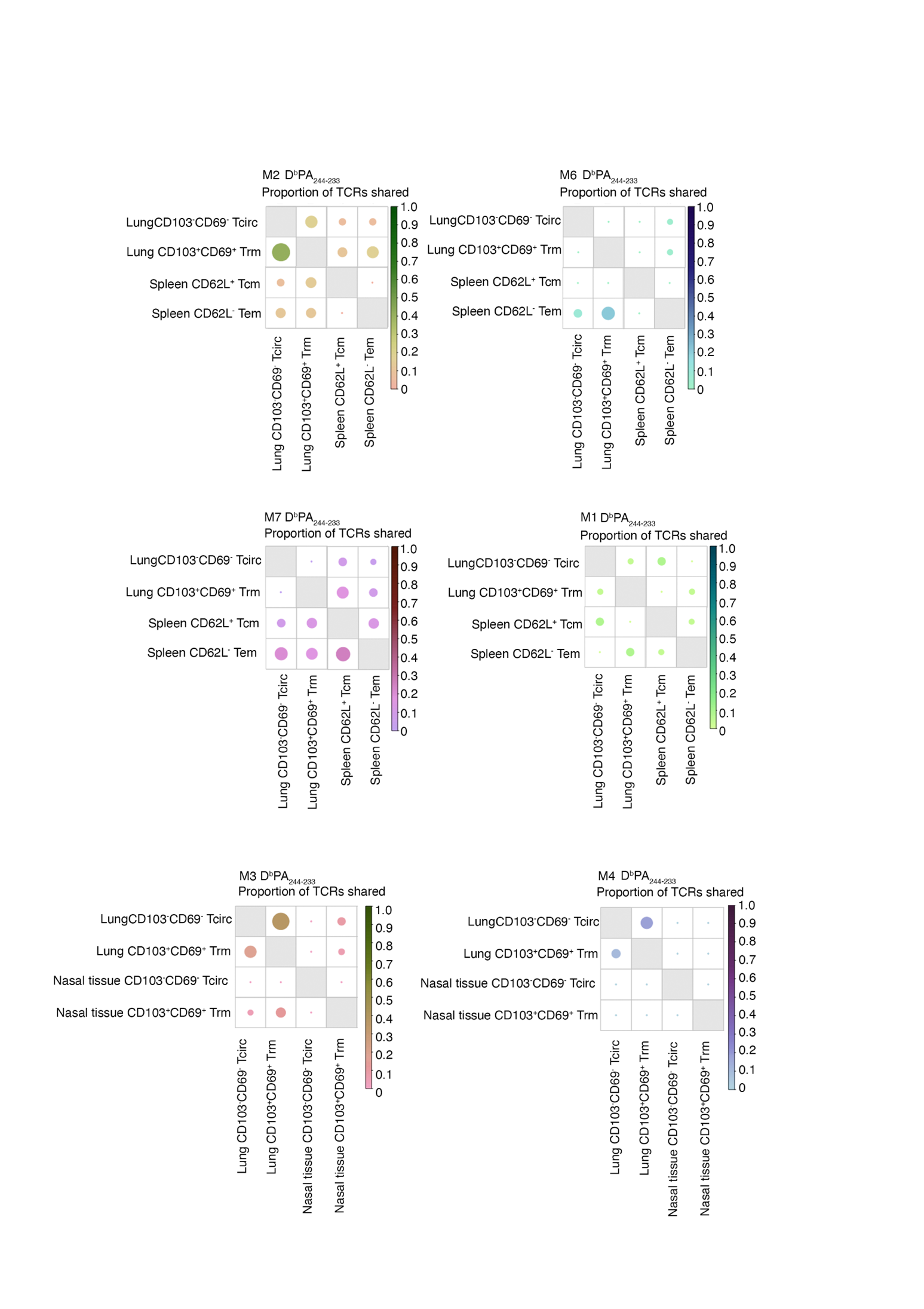


**Supplementary Figure 5:** D^b^**PA_224-233_-specific CD8^+^ T cell TCRαβ clonotype sharing across T cell subset and tissue.**

Proportion of clonotypes shared between D^b^PA_224-233_ tetramer^+^ memory CD8^+^ T cells subsets within individual mice by colour gradient and size of circle.


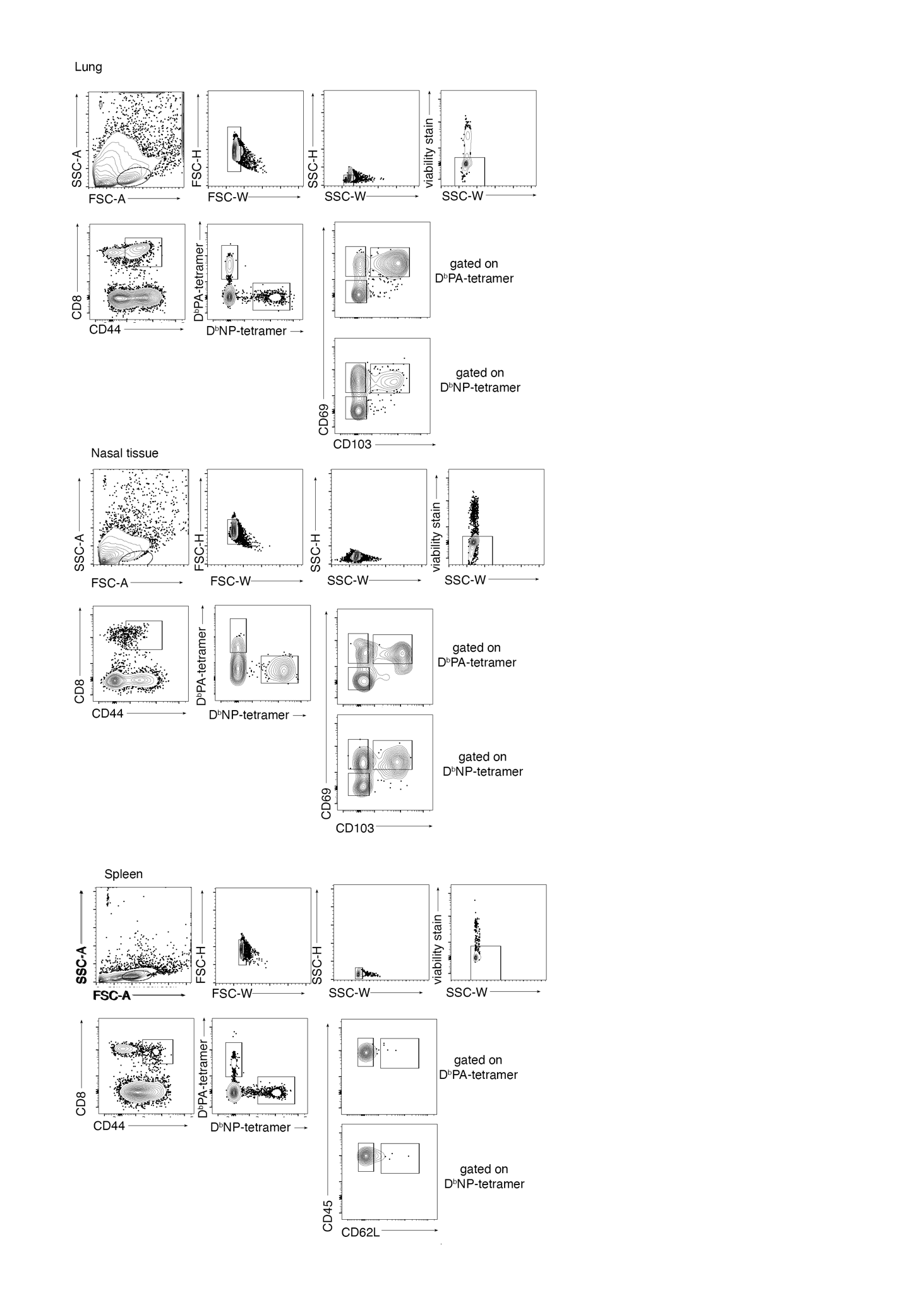


**Supplementary Figure 6: Flow cytometry gating strategies**

Gating strategy for identifying D^b^NP_366_-_374_ and D^b^PA_224_-_233_ specific CD8^+^ T cells in lung, nasal tissue and spleen from mice on day 35 p.i. with X31.
